# How Femoral Neck Resection Height and Dorr Type Affect the Primary Stability of Cemented Short Stems: An In Vitro Study

**DOI:** 10.3390/biology15110826

**Published:** 2026-05-23

**Authors:** Daniel Ch. Haspinger, Stefan Budde, Niels Hammer, Johannes Zeichen

**Affiliations:** 1 Gottfried Schatz Research Center, Division of Macroscopic and Clinical Anatomy, Medical University of Graz, 8036 Graz, Austria; daniel.haspinger@medunigraz.at; 2 Department of Orthopaedic Surgery, Diakovere Annastift, Hannover Medical School, 30625 Hannover, Germany; st-budde@t-online.de; 3 Universitätsklinik für Unfallchirurgie und Orthopädie, Evangelisches Klinikum Bethel, 33617 Bielefeld, Germany; 4 Department of Orthopaedic and Trauma Surgery, University of Leipzig, 04103 Leipzig, Germany; 5 Division of Biomechatronics, Fraunhofer Institute for Machine Tools and Forming Technology (IWU), 01189 Dresden, Germany; 6 Klinik für Unfallchirurgie und Orthopädie, Johannes Wesling Klinikum Minden, 32429 Minden, Germany; johannes.zeichen@muehlenkreiskliniken.de

**Keywords:** biomechanics, calcar-guided short stem, cemented femoral stem, Dorr classification, femoral neck resection height, hip implant stability, in vitro study, primary stability, total hip arthroplasty

## Abstract

Implantation of a femoral stem in total hip arthroplasty alters physiological load transfer within the proximal femur. Short-stem designs aim to preserve bone stock and maintain proximal load sharing, yet the influence of femoral neck resection height and its interaction with femoral morphology on primary stability remain insufficiently understood. This in vitro biomechanical study investigated these effects using 33 human femora classified as Dorr B or C. In a paired design, a cemented calcar-guided short stem was implanted with either a low (standard) or +5 mm higher femoral neck resection. Specimens underwent cyclic fatigue loading to assess reversible and irreversible micromotion and interface strain, followed by ultimate compression to quantify global fixation strength. Primary stability was assessed by reversible and irreversible translation of the prosthetic head center of rotation and by cortical interface strain measurements using digital image correlation. Overall fixation strength and irreversible deformation remained comparable across resection heights and Dorr types. In contrast, resection height and femoral morphology influenced reversible micromotion and interface strain, with higher resection reducing reversible micromotion, particularly in Dorr C femora and shifting lateral interface strain toward compression. These findings suggest that surgical technique and femoral morphology mainly affect local, reversible bone–cement–implant mechanics rather than global fixation strength.

## Simple Summary

Implantation of femoral stems changes how loads are transferred through the proximal femur. Short-stem designs aim to restore more physiological load transfer while preserving bone stock. This biomechanical study investigated how femoral neck resection height and femoral morphology influence micromotion, interface strain, and fixation strength of a cemented short stem under cyclic and failure loading. Using human donor femora and controlled laboratory testing, we found that both factors affect micromotion and strain distribution, although overall implant stability remained comparable. These findings improve the understanding of bone–implant mechanics and may inform future refinement of surgical techniques for cemented short-stem arthroplasty.

## 1. Introduction

The proximal femur is exposed to complex loading during daily activities, including axial compression, bending, and torsion generated by joint contact forces and muscle action. In vivo measurements using instrumented hip implants indicate that hip joint forces typically range from approximately 2–3 times body weight during walking, but may increase to 5–7 times body weight during jogging or stumbling [1,2]. Implantation of a femoral stem in total hip arthroplasty (THA) fundamentally alters the physiological load transfer. Experimental and computational studies demonstrate that femoral stems commonly shift load transfer distally, reducing proximal cortical strains and inducing stress-shielding-related remodeling in the metaphyseal region [3,4,5,6]. Consequently, surgical factors that modify proximal support conditions may influence local stability and load transfer within the bone–implant construct. Because excessive micromotion and unfavorable interface strain may compromise early fixation and contribute to migration or aseptic loosening, these parameters are clinically relevant surrogates of primary stability [7,8].

Short stems have been developed to preserve bone stock, reduce stress shielding, and facilitate potential revision in THA. Many designs aim to achieve physiological load transfer and primary stability while minimizing diaphyseal fixation [9,10,11]. Despite increasing clinical use, the biomechanical consequences of varying femoral neck resection height remain insufficiently understood.

For cementless stems, resection height influences alignment, femoral offset, and initial stability by altering the available bone support [11,12,13]. For cemented stems, however, its biomechanical effect on the bone–cement–implant construct remains unclear. Although the cement mantle can compensate for moderate variations in bone preparation, excessive cortical removal may alter micromotion and strain distribution under load.

Femoral morphology represents another determinant of fixation and stress transmission [14,15]. The Dorr classification describes femoral canal morphology and is widely used as a surrogate indicator of cortical thickness relevant for implant fixation. Dorr C femora, characterized by thin cortices and wide canals, are challenging for cementless fixation and are therefore frequently treated with cemented stems [16,17]. Registry data further indicate an increased risk of periprosthetic fracture with cementless fixation in elderly or osteoporotic bone—morphologies often corresponding to Dorr C femora [18].

To address these challenges, cemented short stems have been introduced. Favorable short-term outcomes have been reported for the cemented A2 short stem (ARTIQO GmbH, Lüdinghausen, Germany), including the absence of aseptic loosening or infection in early follow-up [19]. However, the biomechanical mechanisms by which femoral neck resection height and femoral morphology influence primary stability remain insufficiently quantified in controlled experimental settings, particularly in human donor bone models that realistically reproduce proximal femoral mechanics. To our knowledge, the combined influence of femoral neck resection height and Dorr morphology on local micromotion and interface strain has not been systematically investigated in cemented calcar-guided short stems. This study, therefore, addresses a clinically relevant knowledge gap using human donor femora, cyclic loading, ultimate compression, and digital image correlation-based local strain mapping [12].

The present study investigated the combined effects of femoral neck resection height and Dorr type on the primary stability of a cemented short stem. We hypothesized that primary stability, assessed by reversible and irreversible micromotion, interface strain, and ultimate load capacity, would not differ across resection heights or Dorr types, and that no interaction between these factors would exist.

## 2. Materials and Methods

### 2.1. Specimen Preparation and Classification

Human femora were obtained post-mortem from 19 donors. Specimens were embalmed using a modified Thiel method [20,21,22]. Although Thiel embalming may alter absolute mechanical properties compared with fresh-frozen bone, applying the same fixation protocol to all specimens allowed comparative analyses. Calibrated preoperative radiographs (Ysio FD, Siemens Healthcare GmbH, Erlangen, Germany) were taken to assess femoral morphology using the Dorr classification system [14], determine the planned resection level, and select appropriate implant sizes (Table 1). Radiographs were additionally screened for relevant osseous pathologies such as previous fractures, deformities, severe osteolysis, or prior orthopedic implants that could influence biomechanical testing. Dorr type-B (16 paired, 5 unpaired) and type-C (12 paired) femora were included. After soft tissue removal, specimen length was standardized to 16.5 cm distal to the lesser trochanter. Femoral shafts were then embedded in 3D-printed cups using ceramic-powder-reinforced polyurethane (FC 52/53 Isocyanate and FC 52 Polyol, Huntsman Advanced Materials, Bad Säckingen, Germany; Filler DT082-1, Bodo Möller Chemie GmbH, Offenbach am Main, Germany), ensuring that the diaphyseal axis was perpendicular to the bottom of the cup.

### 2.2. Short-Stem Implantation and Definition of Study Groups

**Figure 1:**
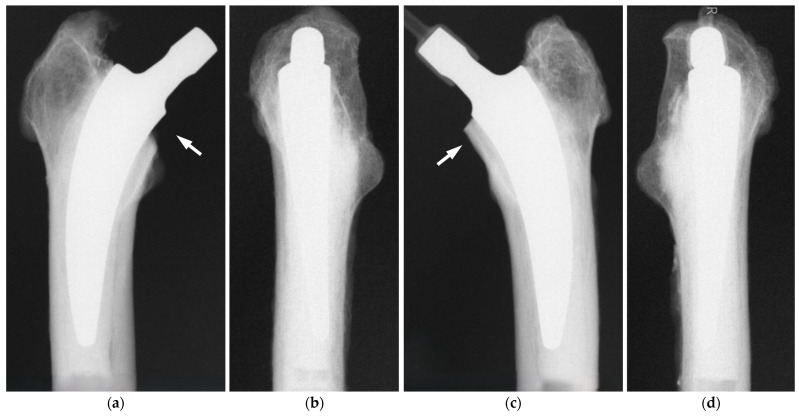
Anteroposterior and lateral radiographs of a pair of femora (individual XV, see Table 1) after implantation of the cemented short stem A2 (ARTIQO GmbH, Lüdinghausen, Germany) with the low resection in the right femur (**a**,**b**) and the high resection in the left femur (**c**,**d**), indicated by white arrows.

Cemented calcar-guided A2 short-stem shafts (ARTIQO GmbH, Lüdinghausen, Germany; manufacturer-defined nominal sizes) were implanted in combination with viscous bone cement (PALACOS^®^, Heraeus Medical GmbH, Wehrheim, Germany) and a bone plug (Cemstop, Teknimed, Bigorre, France). The implantation was performed by two experienced orthopedic and trauma surgeons using the fit-and-fill (FF) technique [10]. Two resection levels were used while preserving the prosthetic head’s center of rotation (Figure 1). The low resection corresponded to the standard resection level recommended for the FF technique. The high resection was defined as a resection performed +5 mm more proximally. Levels were randomly assigned so that each specimen pair contained one of each (Table 1). Correct implant positioning was defined as neutral stem alignment without cortical perforation, as verified on biplanar radiographs (Figure 1).

### 2.3. Biomechanical Testing Setup

**Figure 2:**
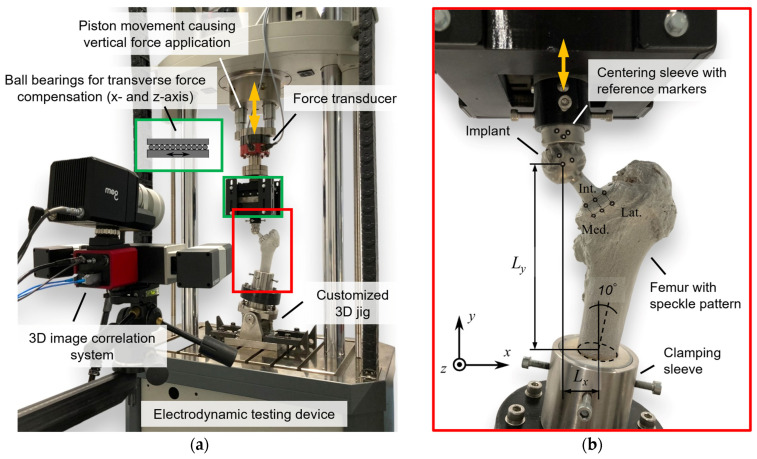
Biomechanical testing setup: (**a**) femur specimen with implanted short stem mounted in the dynamic testing device to simulate single-leg stance using a 3D-jig to apply a 10° adduction and ball bearings to induce purely axial loads; (**b**) speckling pattern and reference markers at pre-defined positions are used to track the current 3D position of the prosthetic head (with respect to the defined cartesian coordinate system) and deformations at the bone–cement–implant interface as well as superficial fractures using an image correlation system.

The femora were mounted in an LTM 10 electrodynamic testing machine (ZwickRoell GmbH & Co. KG, Ulm, Germany) using a custom 3D jig to simulate a single-leg stance (10° adduction, 0° flexion, 0° antetorsion) (Figure 2a). Mechanical loading was applied vertically and concentrically to the prosthetic head via a dynamic force transducer (10 kN; GTM, class 0.5 calibrated), ball bearings to prevent lateral forces, and a cylindrical shaft, following ISO 7206-4 [23] (Figure 2a). Relative motions were recorded using a stereo digital image correlation system (ARAMIS 3D Professional, 12 M cameras, 50 mm lenses, calibration deviation 0.037 pixels; Carl Zeiss GOM Metrology, Braunschweig, Germany), synchronized with the testing device. Six reference markers were symmetrically placed around the resection level to measure strains at lateral, intermediate, and medial positions (Figure 2b). The prosthetic head center of rotation was also tracked via markers. A random speckle pattern was applied to the specimen surface using matte spray paint (European Aerosols GmbH, Haßmersheim, Germany) to monitor the onset and progression of fractures during mechanical testing. For consistency, images of right femora were mirrored so all displacements aligned with the coordinate system in Figure 2b (x: medio-lateral, y: infero-superior, z: postero-anterior).

### 2.4. Investigation of Primary Stability Under Dynamic Loading (Low-Cycle Fatigue Test)

Fatigue strength was evaluated under sinusoidal cyclic loading (2 Hz), beginning at 850 N and increasing by 400 N every 10,000 cycles up to 2.85 kN for a total of 60,000 cycles [24]. This protocol reflects progressively increasing load levels during early postoperative mobilization under controlled conditions. The applied loads correspond to approximately 1–4 times body weight (Table 1), placing them within the range of physiological hip joint forces reported during daily activities such as walking, while remaining below peak loads observed during more demanding activities [1,2]. Dynamic testing was terminated upon vertical head displacement exceeding 5 mm or system failure, defined as any visible fracture gap >1 mm identified visually and verified by digital image correlation. The construct was then unloaded for one hour to mitigate viscoelastic effects and quantify permanent deformation.

The primary stability of the bone–cement–implant system was evaluated using two parameters. Irreversible translation and strain were defined as the residual displacement or strain measured in the unloaded state after completion of the fatigue protocol. Reversible values were calculated as the peak values at cycle 60,000 minus the corresponding irreversible component. Thus, reversible values represent load-dependent deformation during cyclic loading, whereas irreversible values reflect permanent deformation remaining after unloading. Negative strain values indicate compressive deformation, whereas positive values represent tensile deformation.

### 2.5. Quantification of Mechanical Failure Resistance (Ultimate Compression Test)

For all specimens surviving the low-cycle fatigue test without failure, an ultimate compression test was performed [24]. A 100 N/s linearly increasing load was applied until failure, defined as a >30% force drop from the maximum. To prevent overload, a 13 kN limit was set for the force transducer. The fracture properties were analyzed using the peak load, its corresponding head translation, and interface strain.

### 2.6. Data Analysis

All outcome parameters were computed using Matlab R2023b (The MathWorks Inc., Natick, MA, USA) and GOM Correlate Professional 2020 (Carl Zeiss GOM Metrology, Braunschweig, Germany). Statistical analyses and graphical visualization were performed in Python version 3.8.7 (Python Software Foundation, Wilmington, NC, USA) and GraphPad Prism version 10.4.1 (Boston, MA, USA).

Normality (Shapiro–Wilk) and homogeneity of variance (Levene) were assessed prior to inferential testing. Main and interaction effects of femoral morphology (Dorr type B/C) and resection height (high/low) were analyzed using two-way ANOVA or Scheirer–Ray–Hare tests where appropriate. Group comparisons were performed using unpaired *t*-tests or Mann–Whitney U tests.

Due to the experimental design, a partially paired dataset was obtained, as not all specimens could be tested under both resection conditions. Therefore, statistical analyses were primarily conducted using independent group comparisons. However, paired allocation was maintained during specimen preparation to minimize inter-individual variability and ensure balanced group characteristics. The potential influence of pairing was considered during interpretation of the results.

Primary analyses focused on main and interaction effects, whereas subgroup analyses were considered exploratory and hypothesis-generating. Given the hypothesis-driven nature of the primary comparisons and the limited sample size, no formal multiple-testing correction was applied to avoid inflation of type II error. To account for the associated risk of type I error, results were interpreted in conjunction with effect sizes and consistency across related outcome measures.

Due to the limited availability of human donor femora suitable for biomechanical testing, an a priori power analysis was not feasible. The study should therefore be interpreted as exploratory, particularly with respect to subgroup analyses. Post hoc power analyses were performed for significant findings (α = 0.05) to describe the achieved statistical power, but were not used as a substitute for prospective sample size justification. Effect sizes were calculated (η_p_^2^ for ANOVA, ε^2^ for Scheirer–Ray–Hare tests, Cohen’s d for *t*-tests, and Cliff’s δ for Mann–Whitney U tests) and interpreted using conventional thresholds [25]: small, medium, large effects correspond to η_p_^2^/ε^2^ ≥ 0.01, ≥0.06, ≥0.14; Cohen’s d ≥ 0.2, ≥0.5, ≥0.8; and Cliff’s δ ≥ 0.147, ≥0.33, ≥0.474.

Potential associations between testing parameters and biomechanical outcomes were explored using Pearson or Spearman correlation analyses, including correlations between clamping length and outcome parameters to assess potential boundary-condition effects related to body donor height (Table 1).

## 3. Results

### 3.1. Overall Specimen Performance

Mechanical testing confirmed stable construct performance. A total of 33 femora from 19 donors were tested. All but one specimen completed the 60,000-cycle fatigue protocol without detectable superficial fractures. During ultimate compression, three specimens reached the 13-kN device limit without fracture and were included in the analysis.

Primary analyses focused on main and interaction effects of resection height and Dorr type, whereas subgroup analyses were considered exploratory and interpreted accordingly.

### 3.2. Resection Height Governs Reversible Biomechanics and Load Transfer

**Figure 3:**
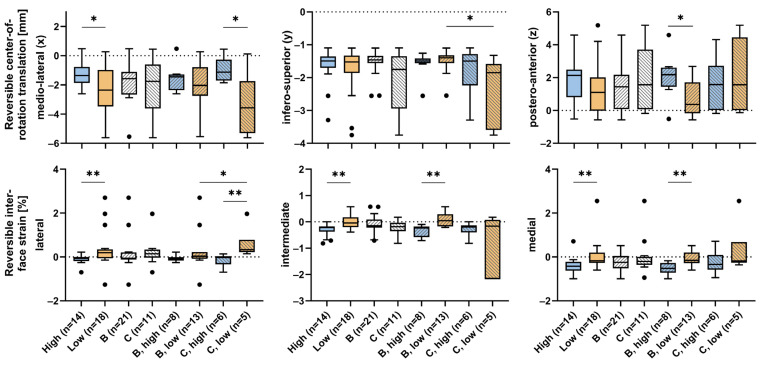
Reversible prosthetic head translation and interface strain after low-cycle fatigue testing. Tukey boxplots show translation in the medio-lateral, infero-superior, and postero-anterior directions (**top** row) and interface strain at lateral, intermediate, and medial positions (**bottom** row). Main differences were observed in medio-lateral translation and lateral interface strain. Fill color indicates resection height; hatching indicates Dorr type and subgroup allocation. Asterisks denote statistical significance (* *p* ≤ 0.05, ** *p* ≤ 0.01).

Femoral neck resection height was a primary determinant of biomechanical behavior, significantly reducing reversible medio-lateral head translation with high resection (η_p_^2^ = 0.15, *p* = 0.030) and associated with a qualitative shift in load-transfer mechanics at the implant interface (Figure 3, Table A1). This was reflected by a shift in the lateral strain pattern: high resection induced compressive (negative) strains, while low resection produced tensile (positive) strains, a difference with a large effect size (ε^2^ = 0.20, *p* = 0.007). The altered load transfer was also reflected at other interface positions: high resection also produced larger reversible strains at the intermediate (ε^2^ = 0.22, *p* = 0.004) and medial (ε^2^ = 0.21, *p* = 0.006) locations, both large effects.

**Figure 4:**
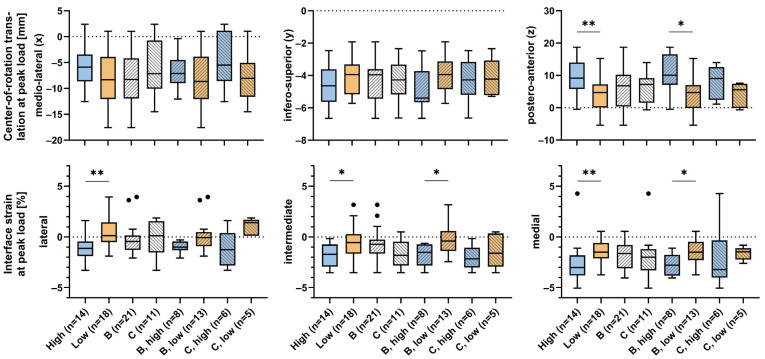
Prosthetic head translation and interface strain at peak load by resection height (high, low) and Dorr type (B, C). Tukey boxplots show translation in the medio-lateral, infero-superior, and postero-anterior directions (**top** row) and interface strain at lateral, intermediate, and medial positions (**bottom** row). Differences were mainly observed in postero-anterior translation and interface strain, while medio-lateral and infero-superior translation remained comparable. Fill color indicates resection height, and hatching indicates Dorr type and subgroup allocation. Asterisks denote statistical significance (* *p* ≤ 0.05, ** *p* ≤ 0.01).

At peak load, the influence of resection height was also apparent. While medio-lateral and infero-superior head displacement were unaffected, high resection resulted in greater postero-anterior displacement, a difference with a large effect size (η_p_^2^ = 0.24, *p* = 0.006). Furthermore, at peak load, the high resection group exhibited larger compressive interface strains at the lateral (ε^2^ = 0.23, *p* = 0.006), intermediate (η_p_^2^ = 0.16, *p* = 0.03), and medial (ε^2^ = 0.21, *p* = 0.005) positions, all large effects (Figure 4, Table A2).

### 3.3. Bone Morphology Modulates the Response to Surgical Technique

Bone morphology showed no main effect but interacted with resection height to influence reversible interface strains, with large effect sizes at the lateral (ε^2^ = 0.34, *p* = 0.008), intermediate (ε^2^ = 0.31, *p* = 0.012), and medial (ε^2^ = 0.25, *p* = 0.028) positions (Figure 3). A large interaction effect was also found for strains at peak load at the lateral (ε^2^ = 0.29, *p* = 0.020) and medial (ε^2^ = 0.22, *p* = 0.050) locations, showing that the effect of surgical technique depended on femoral morphology (Figure 4).

Exploratory subgroup analyses provided further insight into this interaction, although results should be interpreted with caution due to limited sample sizes: biomechanical effects of resection height appeared more pronounced in Dorr C femora. For example, the reduction in reversible medio-lateral translation with a high resection was observed only in Dorr C specimens, with a large effect (Cohen’s d = 1.50, *p* = 0.026). In contrast, Dorr B femora exhibited smaller postero-anterior translation in the low compared to the high resection group, also a large-effect difference (Cohen’s d = 1.1, *p* = 0.027). The influence of resection height on interface strains also varied by bone type. In Dorr B femora, low resection resulted in smaller reversible strains at the intermediate (Cliff’s δ = 0.82, *p* = 0.002) and medial position (Cliff’s δ = 0.69, *p* = 0.008). In contrast, for Dorr C femora, the most pronounced difference was at the lateral interface, where low resection produced substantially larger tensile strains than high resection (Cliff’s δ = 1.0, *p* = 0.002).

Furthermore, within the low resection group, Dorr C femora exhibited significantly greater reversible infero-superior translation (Cliff’s δ = 0.59, *p* = 0.046) and lateral interface strain (Cliff’s δ = 0.62, *p* = 0.037) than Dorr B femora, with both differences showing a large effect. These differences between Dorr types were absent in the high resection group, suggesting that high resection may be associated with more comparable biomechanical behavior in femora with reduced cortical support (Figure 3, Table A1).

At peak load, subgroup analyses showed consistent patterns with the reversible data: in Dorr B femora, high resection resulted in greater postero-anterior translation compared to low resection (Cohen’s d = 1.2, *p* = 0.02), accompanied by larger interface strains at the intermediate (Cohen’s d = 1.1, *p* = 0.03) and medial positions (Cliff’s δ = 0.60, *p* = 0.02). These findings were consistent with the reversible behavior, suggesting that resection height also influenced load transfer during peak loading.

Overall, these subgroup findings consistently supported the observed interaction patterns but should be interpreted as exploratory.

### 3.4. Irreversible Stability and Peak Load Are Independent of Resection Height and Dorr Type

**Figure 5:**
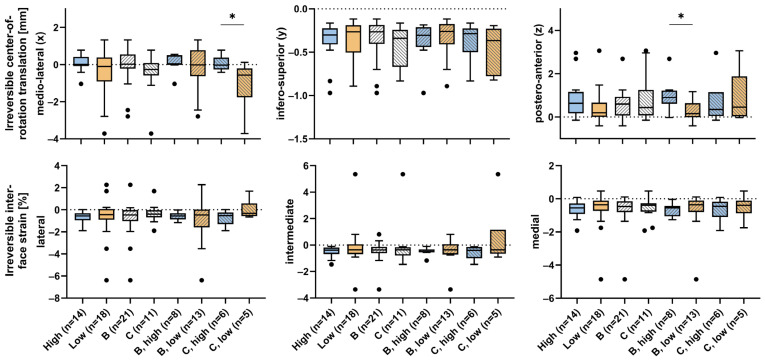
Irreversible prosthetic head translation and interface strain after low-cycle fatigue testing. Tukey boxplots show residual translation in the medio-lateral, infero-superior, and postero-anterior directions (**top** row) and residual interface strain at lateral, intermediate, and medial positions (**bottom** row). No consistent main effects of resection height (high, low) or Dorr type (B, C) were observed, indicating comparable irreversible stability across groups. Fill color indicates resection height, and hatching indicates Dorr type and subgroup allocation. Asterisks denote statistical significance (* *p* ≤ 0.05).

Importantly, there were no differences in irreversible head translations or interface strains between resection groups or Dorr types, indicating consistent global stability across all groups (Figure 5, Table A1). Subgroup analyses, however, revealed isolated exploratory differences: in Dorr C femora, high resection resulted in lower medio-lateral translation compared to low resection (Cliff’s δ = 0.72, *p* = 0.041), whereas in Dorr B femora, postero-anterior translation was reduced with low compared to high resection (Cliff’s δ = 0.62, *p* = 0.02).

**Figure 6:**
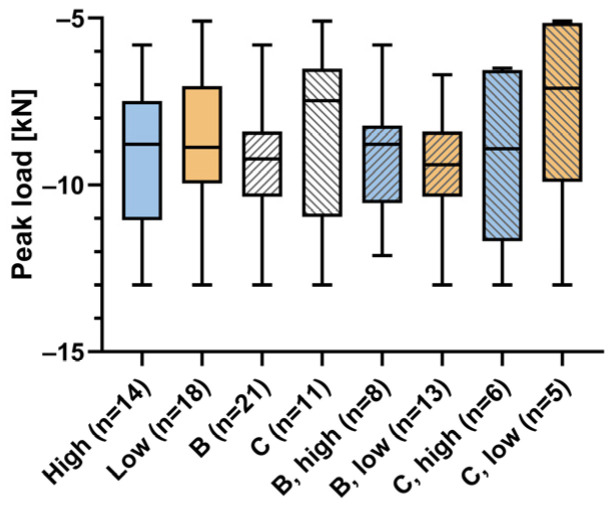
Ultimate compression load by resection height (high, low) and Dorr type (B, C). Tukey boxplots show the maximum load achieved during ultimate compression testing. Peak load did not differ between resection heights or Dorr types, indicating comparable global fixation strength across groups. Fill color indicates resection height, and hatching indicates Dorr type and subgroup allocation.

Ultimate peak load capacity did not differ between groups, indicating robust fixation strength regardless of surgical technique or bone morphology (Figure 6, Table A2).

### 3.5. Ancillary Data and Correlations

There were no significant differences in demographic data, implanted stem sizes, or clamping lengths between groups. This applied to both primary group comparisons (Dorr B vs. C: all *p* ≥ 0.13; high vs. low resection: all *p* ≥ 0.07) and all subgroup comparisons (all *p* ≥ 0.06), confirming appropriate specimen allocation (Table 1). As an internal validation, several testing parameters correlated with outcomes. Notably, lateral clamping length (L_x_) correlated strongly with peak load (Pearson’s r = 0.625, *p* < 0.001) and moderately with COR translations at peak load in the medio-lateral (Pearson’s r = −0.426, *p* = 0.013) and infero-superior (Pearson’s r = −0.423, *p* = 0.014) directions. Longitudinal clamping length (L_y_) also correlated with intermediate interface strain at peak load (Pearson’s r = −0.373, *p* = 0.033). These correlations were observed across all specimens and were not driven by group differences.

## 4. Discussion

This in vitro study shows that a cemented calcar-guided short stem achieved robust global primary stability across resection heights and Dorr morphologies. Ultimate load capacity and irreversible deformation remained comparable between groups, indicating that neither resection height nor Dorr type substantially altered global fixation strength under the present loading conditions. In contrast, resection height and femoral morphology influenced local, predominantly reversible micromotion and interface strain. These findings suggest that surgical technique primarily affects load-dependent bone–cement–implant mechanics rather than ultimate fixation strength.

The clearest local finding was the shift in lateral strain: low resection produced net tensile strains, whereas high resection shifted the lateral interface toward compression. This is biomechanically relevant because compressive loading can increase frictional resistance and support load sharing at the bone–cement–implant interface, whereas tensile loading may promote interface opening and micromotion, both of which have been linked to migration and aseptic loosening [26,27,28,29]. The loading state of the proximal femur remains debated, with models predicting either lateral tension from bending or more generalized compression when muscle forces are included [30]. The present findings indicate that femoral neck resection height can modify this local loading environment.

Mechanistically, the strain reversal may be explained by altered proximal support conditions. A calcar-guided short stem relies on cortical apposition and load transfer via the femoral calcar. A higher resection preserves more calcar-supported cortical bone, potentially creating a medial buttress that limits subsidence and varus toggling under axial loading [10,12]. This may promote lateral compressive reaction forces and a more favorable load-sharing configuration. In contrast, a lower resection reduces medial support and may allow greater bending-related tensile strains at the lateral interface.

The morphology-dependent response was most apparent in Dorr C femora. Thin cortices and wide canals may amplify the mechanical consequences of altered proximal support, making local strain patterns more sensitive to resection height [15]. In this morphology, low resection was associated with greater lateral tensile strain and reversible micromotion, whereas high resection shifted the local strain state toward compression. Dorr B femora, with thicker cortices and narrower canals, appeared less sensitive to resection height, which may explain why local differences were smaller and partly location- or direction-dependent.

The observed sensitivity of local strain patterns to resection height is consistent with previous experimental work showing that femoral neck resection height can alter proximal femoral strain patterns in short-stem arthroplasty, although that study used a different cementless short-stem concept and composite femora [31]. Previous in vitro work on cemented short stems also reported favorable primary stability and fracture resistance [24]. However, available evidence on short-stem primary stability remains limited to relatively few implant designs [7], and cemented short stems have been investigated far less extensively than cementless short stems [32]. To our knowledge, the combined effect of resection height and Dorr morphology on local micromotion and interface strain in cemented calcar-guided short stems has not been systematically investigated. The present study extends previous evidence by showing that resection height can modify local bone–cement–implant mechanics in a morphology-dependent manner, while global fixation strength remained comparable.

These findings are also consistent with the rationale for cemented fixation in femora with reduced cortical support. Registry-based evidence indicates a lower fracture risk with cemented fixation in elderly and osteoporotic populations compared with cementless fixation [18], and Dorr C morphology is commonly considered challenging for cementless stem fixation [14,15,16,17]. The present data add a local biomechanical perspective by showing that the cemented short-stem construct maintained comparable ultimate load capacity and irreversible stability even in Dorr C femora, while local reversible strain patterns remained sensitive to resection height.

Short stems were originally developed mainly to preserve bone stock and reduce stress shielding, concepts most commonly associated with cementless fixation [33]. The cemented calcar-guided short stem combines a bone-preserving stem concept with cemented fixation, which may be relevant for older or morphologically compromised femora. Favorable early clinical outcomes have been reported for the cemented A2 short stem [19]. However, the present study should be interpreted as a biomechanical investigation of primary stability and local load transfer rather than as evidence of clinical superiority.

From a technical perspective, these findings suggest that femoral neck resection height may be particularly relevant in Dorr C morphology. A resection approximately 5 mm more proximal was associated with reduced reversible medio-lateral micromotion and a shift in lateral interface strain toward compression, without increasing irreversible deformation or reducing ultimate load capacity. This observation may inform surgical planning, but it should not be interpreted as a direct clinical recommendation because the relevant findings were local, predominantly reversible, and partly derived from exploratory subgroup analyses.

In Dorr B femora, resection height appeared less critical for global primary stability. Although some direction-dependent reversible differences were observed, ultimate load capacity and irreversible deformation remained comparable. Therefore, in femora with more favorable cortical support, the biomechanical constraints related to resection height may be less pronounced, potentially allowing reconstruction parameters such as leg length, offset, and center of rotation to receive greater consideration. These technique-related considerations require validation in larger experimental studies and prospective clinical investigations.

These findings should be considered within the study’s methodological context. Strengths include the paired human donor design, the clinically relevant fatigue-then-failure protocol [24], and high-resolution digital image correlation for local strain and motion mapping.

The primary limitation is the use of Thiel-embalmed specimens, which may alter bone mechanics compared with fresh-frozen tissue [21]. Therefore, absolute load and deformation values should be interpreted with caution. At the same time, the experimental design enables controlled assessment of proximal femoral bone–implant mechanics that cannot be reproduced in vivo. Because all specimens followed the same preparation and testing protocol, comparisons between resection heights and Dorr morphologies remain interpretable.

A second limitation is the simplified axial loading configuration, which does not reproduce the full in vivo loading environment, including torsional moments, muscle forces, and time-dependent biological remodeling. Therefore, potential failure modes such as rotational instability and long-term adaptive bone remodeling could not be assessed. Nevertheless, the loading configuration represents a simplified but widely used approximation of single-leg stance conditions in biomechanical testing of femoral stems according to ISO 7206-4 [23].

Clamping length did not differ between groups, suggesting no systematic bias; however, it correlated with outcome parameters across specimens, indicating that the experimental setup was sensitive to boundary conditions. Subgroup analyses had limited power (e.g., *n* = 5 for Dorr C with low resection), and the distribution of sex across Dorr types may have introduced residual confounding, as sex is associated with bone mineral density and proximal femoral geometry [16,34]. Therefore, Dorr-related effects should be interpreted as morphology-associated findings rather than isolated effects of bone quality. Larger studies with balanced sex distribution and quantitative bone-density assessment are needed for confirmation.

Furthermore, cement mantle thickness and distribution were not quantified in this study. Although standardized implantation techniques were used, variations in cement mantle geometry may influence load transfer and micromotion at the bone–cement interface.

## 5. Conclusions

This study demonstrates robust global primary stability of a cemented calcar-guided short stem across resection heights and Dorr types. Resection height and femoral morphology mainly affected local, predominantly reversible micromotion and interface strain, while ultimate load capacity and irreversible deformation remained comparable. In Dorr C femora, a resection at +5 mm was associated with reduced reversible medio-lateral micromotion and a shift in lateral interface strain toward compression. These findings suggest that morphology-dependent resection height may influence local bone–cement–implant mechanics in cemented short-stem arthroplasty.

## Figures and Tables

**Table 1:** Donor characteristics and specimen allocation, including Dorr type classification, femoral neck resection level (H = high resection (+5 mm above the low resection height); L = low resection), and manufacturer-defined nominal implant size (ARTIQO GmbH, Lüdinghausen, Germany). Thirty-three femora from 19 donors were investigated (10 male, 9 female; Dorr B/C = 13/6). Continuous variables are reported as median (interquartile range): age 79 (74.5–89.5) years, height 170 (160–177) cm, weight 70 (59.5–86) kg. Implant size: left 5 (4–6), right 5 (5–6).

Individual	Sex	Age [y]	Height [cm]	Weight [kg]	Dorr Type	Resection Level (Left/Right)	Implant Size (Left/Right)
I	m	71	190	130	B	H/L	5/5
II	f	92	158	55	B	H/L	4/4
III	f	78	162	72	B	H/L	4/4
IV	m	93	171	47	B	L/–	9/–
V	m	76	173	70	B	–/L	–/5
VI	f	79	160	62	B	H/L	4/5
VII	f	58	166	54	B	H/L	4/5
VIII	m	95	180	90	B	H/L	7/8
IX	m	79	174	67	B	H/L	6/6
X	m	77	179	90	B	–/L	–/3
XI	m	92	170	75	B	H/L	5/6
XII	m	80	175	66	B	L/–	6/–
XIII	m	79	170	64	B	L/–	5/–
XIV	f	87	160	57	C	H/L	7/7
XV	f	73	156	70	C	H/L	5/5
XVI	m	76	180	95	C	H/L	7/7
XVII	f	58	182	91	C	H/L	4/5
XVIII	f	96	152	40	C	H/L	5/6
XIX	f	70	160	82	C	H/L	5/6

## Data Availability

The data that support the findings of this study are available from the corresponding author upon reasonable request.

## Abbreviations

The following abbreviations are used in this manuscript:

THA	Total hip arthroplasty
FF	Fit-and-fill
COR	Center of rotation
H	High
L	Low

## Appendix A

**Table A1:** Reversible and irreversible prosthetic head center-of-rotation (COR) translations and interface strains after low-cycle fatigue loading (60,000 cycles with a maximum load of 2.85 kN). Values are reported as median (interquartile range). Negative values indicate compressive loading.

Group	Reversible COR Translation [mm]			Reversible Interface Strain [%]		
	Medio- Lateral (x)	Infero- Superior (y)	Postero- Anterior (z)	Lateral	Intermediate	Medial
High (*n* = 14)	−1.4 (−1.9–−0.8)	−1.5 (−1.7–−1.4)	2.1 (0.8–2.5)	−0.06 (−0.19–0.00)	−0.21 (−0.37–−0.17)	−0.43 (−0.62–−0.22)
Low (*n* = 18)	−2.4 (−3.5–−1.0)	−1.5 (−1.9–−1.3)	1.1 (0.0–2.0)	0.19 (−0.06–0.35)	−0.05 (−0.20–0.18)	−0.17 (−0.28–0.20)
B (*n* = 21)	−1.6 (−2.7–−1.1)	−1.5 (−1.6–−1.3)	1.4 (0.1–2.2)	−0.06 (−0.12–0.21)	−0.14 (−0.22–0.09)	−0.25 (−0.52–0.04)
C (*n* = 11)	−1.8 (−3.6–−0.6)	−1.8 (−2.9–−1.3)	1.6 (0.1–3.7)	0.14 (−0.03–0.34)	−0.18 (−0.36–−0.03)	−0.21 (−0.36–0.01)
B, high (*n* = 8)	−1.4 (−2.4–−1.3)	−1.5 (−1.6–−1.4)	2.2 (1.4–2.6)	−0.09 (−0.16–−0.01)	−0.23 (−0.58–−0.18)	−0.52 (−0.72–−0.28)
B, low (*n* = 13)	−2.0 (−2.8–−0.8)	−1.4 (−1.6–−1.3)	0.4 (−0.2–1.7)	0.03 (−0.08–0.22)	0.04 (−0.17–0.29)	−0.15 (−0.28–0.20)
C, high (*n* = 6)	−1.1 (−1.7–−0.3)	−1.5 (−2.2–−1.3)	1.6 (0.0–2.7)	−0.02 (−0.34–0.04)	−0.19 (−0.40–−0.13)	−0.35 (−0.58–0.09)
C, low (*n* = 5)	−3.6 (−5.3–−1.7)	−1.9 (−3.6–−1.6)	1.6 (0.0–4.4)	0.33 (0.22–0.78)	−0.16 (−2.18–0.08)	−0.18 (−0.26–0.68)
**Group**	**Irreversible COR Translation [mm]**			**Irreversible Interface Strain [%]**		
	**Medio-** **Lateral (x)**	**Infero-** **Superior (y)**	**Postero-** **Anterior (z)**	**Lateral**	**Intermediate**	**Medial**
High (*n* = 14)	0.0 (−0.1–0.4)	−0.3 (−0.4–−0.2)	0.6 (0.2–1.2)	−0.56 (−0.96–−0.31)	−0.39 (−0.68–−0.18)	−0.55 (−0.91–−0.28)
Low (*n* = 18)	−0.1 (−0.9–0.4)	−0.3 (−0.5–−0.2)	0.2 (0.0–0.7)	−0.43 (−0.90–0.07)	−0.36 (−0.69–0.06)	−0.36 (−0.73–−0.11)
B (*n* = 21)	0.0 (−0.2–0.5)	−0.3 (−0.4–−0.2)	0.6 (0.1–0.9)	−0.47 (−1.04–−0.03)	−0.37 (−0.63–−0.14)	−0.46 (−0.80–−0.14)
C (*n* = 11)	−0.3 (−0.6–0.1)	−0.3 (−0.7–−0.2)	0.4 (0.1–1.3)	−0.39 (−0.69–−0.04)	−0.36 (−0.79–−0.16)	−0.39 (−0.78–−0.26)
B, high (*n* = 8)	0.0 (0.0–0.5)	−0.3 (−0.4–−0.2)	0.9 (0.6–1.2)	−0.56 (−0.87–−0.30)	−0.39 (−0.55–−0.33)	−0.55 (−1.06–−0.43)
B, low (*n* = 13)	0.0 (−0.6–0.8)	−0.3 (−0.4–−0.2)	0.2 (0.0–0.6)	−0.47 (−1.59–0.02)	−0.36 (−0.71–0.07)	−0.36 (−0.80–−0.10)
C, high (*n* = 6)	0.0 (−0.3–0.4)	−0.3 (−0.5–−0.2)	0.3 (0.0–1.2)	−0.52 (−1.29–−0.24)	−0.41 (−1.00–−0.15)	−0.46 (−1.10–−0.17)
C, low (*n* = 5)	−0.6 (−1.8–−0.2)	−0.4 (−0.8–−0.2)	0.5 (0.0–1.9)	−0.32 (−0.57–0.58)	−0.36 (−0.66–1.15)	−0.39 (−0.87–−0.11)

**Table A2:** Peak load, corresponding prosthetic head center-of-rotation (COR) translations, and interface strains at three pre-defined positions. Values are reported as median (interquartile range). Negative values indicate compressive loading.

Group	Peak Load [kN]	Spatial COR Translation [mm]			Interface Strain [%]		
		Medio- Lateral (x)	Infero- Superior (y)	Postero- Anterior (z)	Lateral	Intermediate	Medial
High (*n* = 14)	−8.8 (−11.0–−7.5)	−5.9 (−8.6–−3.4)	−4.6 (−5.6–−3.6)	9.1 (5.8–14.0)	−1.1 (−1.9–−0.4)	−1.7 (−2.9–−0.7)	−3.0 (−3.8–−1.8)
Low (*n* = 18)	−8.9 (−10.0–−7.0)	−8.3 (−12.0–−3.9)	−3.9 (−5.2–−3.3)	4.7 (0.1–7.2)	0.1 (−0.5–1.4)	−0.6 (−1.7–0.3)	−1.5 (−2.1–−0.6)
B (*n* = 21)	−9.2 (−10.0–−8.4)	−8.3 (−12.0–−4.2)	−4.0 (−5.4–−3.6)	6.8 (0.4–10.0)	−0.5 (−1.3–0.1)	−0.7 (−1.7–−0.3)	−1.6 (−3.1–−0.8)
C (*n* = 11)	−7.5 (−11.0–−6.5)	−7.2 (−10.0–−0.7)	−4.3 (−5.2–−3.3)	7.2 (1.5–9.1)	0.1 (−1.5–1.5)	−1.8 (−2.8–−0.5)	−2.0 (−3.3–−1.2)
B, high (*n* = 8)	−8.8 (−11.0–−8.2)	−7.1 (−9.0–−4.5)	−5.4 (−5.7–−3.7)	10.0 (7.1–17.0)	−1.0 (−1.3–−0.4)	−1.5 (−2.8–−0.7)	−2.8 (−3.8–−1.8)
B, low (*n* = 13)	−9.4 (−10.0–−8.4)	−8.7 (−12.0–−3.9)	−3.9 (−4.8–−3.1)	4.7 (−0.2–7.0)	−0.1 (−0.9–0.5)	−0.4 (−1.4–0.6)	−1.5 (−2.3–−0.5)
C, high (*n* = 6)	−8.9 (−12.0–−6.6)	−5.5 (−8.6–1.2)	−4.3 (−5.2–−3.2)	9.0 (2.4–13.0)	−1.3 (−2.8–0.4)	−2.2 (−3.0–−1.1)	−3.2 (−4.0–−0.3)
C, low (*n* = 5)	−7.1 (−9.9–−5.1)	−8.1 (−12.0–−5.0)	−4.2 (−5.2–−3.1)	5.6 (−0.1–7.4)	1.4 (0.1–1.7)	−1.6 (−2.9–0.3)	−1.5 (−2.2–−1.1)
